# Reviewer acknowledgement

**DOI:** 10.1186/1756-6606-6-7

**Published:** 2013-03-07

**Authors:** Tim Bliss, Bong-Kiun Kaang, Min Zhuo

**Affiliations:** 1 Department of Biological Sciences, Seoul National University, Seoul, Korea

## Abstract

**Contributing reviewers:**

The editors of Molecular Brain would like to thank all our reviewers who have contributed to the journal in Volume 5 (2012).

## 

David Adams

Australia

Hiroshi Ageta

Japan

Candice Askwith

United States of America

Inna Belfer

United States of America

Peter Bergold

United States of America

Vadim Bolshakov

United States of America

Hong Cao

China

Yaomin Chen

United States of America

Eunji Cheong

Korea, South

Se-Young Choi

Korea, South

Graham Collingridge

United Kingdom

Nigel Emptage

United Kingdom

Zhong-Ping Feng

Canada

Stephen Ferguson

Canada

Paul Fisher

United States of America

Paul Frankland

Canada

Yu Fu

United States of America

Yong-Jing Gao

China

Yingbo He

United States of America

Sheng Hou

Canada

Andrew Huberman

United States of America

Kaoru Inokuchi

Japan

Etsuro Ito

Japan

Robert Jensen

United States of America

Guangchen Ji

United States of America

Sheena Josselyn

Canada

Oksana Kaidanovich-Beilin

Canada

Brad Keele

United States of America

Satoshi Kida

Japan

Yang Kim

Korea, South

Byung Gon Kim

Korea, South

Eunjoon Kim

Korea, South

Joung Hun Kim

Korea, South

Masami Kojima

Japan

Sung Joong Lee

Korea, South

C. Justin Lee

Korea, South

Daewoo Lee

United States of America

Kyungmin Lee

Korea, South

Jang-Hern Lee

Korea, South

Jin-A Lee

Korea, South

Meng Li

United Kingdom

Weidong Li

United States of America

Kaicheng Li

China

Xiang-Yao Li

China

Xiao-Jiang Li

United States of America

Yun-Qing Li

China

Wei-Yang Lu

Canada

Masabumi Minami

Japan

Tsuyoshi Miyakawa

Japan

Hisashi Mori

Japan

Henry Mosberg

United States of America

Jose R. Naranjo

Spain

Pasquale Niscola

Italy

Tomohiro Numata

Japan

Hideyuki Okano

Japan

Klaus Reymann

Germany

Jeongseop Rhee

Germany

Jumpei Sasabe

Japan

Noriaki Sasai

United Kingdom

Frederic Saudou

France

Alcino Silva

United States of America

Terrance Snutch

Canada

Jihwan Song

Korea, South

Wayne Sossin

Canada

Judith Strong

United States of America

Leigh Anne Swayne

Canada

Roger Thompson

Canada

Hiroki Toyoda

Japan

Hiroshi Ueda

Japan

Annamaria Vezzani

Italy

Yun Wang

China

Lu-Yang Wang

Canada

Long-Jun Wu

United States of America

Dimitris Xanthos

Austria

Jun Xia

Hong Kong

Fuqiang Xu

China

Jun Yan

Canada

Zhiqiang Yan

United States of America

Bing Ye

United States of America

Megumu Yoshimura

Japan

Lei Yu

United States of America

Gerald W Zamponi

Canada

Shaoqun Zeng

China

